# High Frequency Currents

**Published:** 1907-09

**Authors:** 


**High Frequency Currents.** By H. Evelyn Crook, M.D., B.S. Pp. x., 206. London : Baillière, Tindall and Cox. 1907.

There can be no doubt that in certain forms of disease treatment by the high frequency electrical currents is of very great value, and it is in the hope "that it may in some way help to bring before the notice of medical men the undoubted therapeutical value of high frequency currents in certain pathological conditions," that the author submits this book to the medical profession.

The first part of the book is devoted to a description of the production of the currents, and of the various forms of apparatus before the public for the purpose. This description is most thorough, and moreover is given in a most lucid style, the result being that it is one of the most valuable and helpful accounts that we have met with.

The second part is devoted to the consideration of the physiological effects of the currents, in which a very good summary of the work of the best observers—more especially on the continent —is given.

The rest of the book is given up to the consideration of the therapeutical uses of high frequency currents, and it is this part that will probably be of most interest to the practitioner. It mainly consists of reports of cases by various workers, which prove undoubtedly that in tuberculous disease, rheumatic disease of all kinds, &c., the high frequency currents are of very great value.

This form of electrical treatment has perhaps suffered from too much being expected of it when it was first introduced, and also, perhaps, as is pointed out in the preface, " because it has unfortunately in many instances fallen into the hands of irresponsible and unqualified persons ; " nevertheless, it is of the greatest use in certain forms of disease, and such treatises as the one under review, written in a dispassionate and judicial way, will do much to place the treatment on its proper footing in therapeutics.

